# How do hospitalised patients with Turkish migration background estimate their language skills and their comprehension of medical information – a prospective cross-sectional study and comparison to native patients in Germany to assess the language barrier and the need for translation

**DOI:** 10.1186/1472-6963-13-196

**Published:** 2013-05-28

**Authors:** Arnd Giese, Müberra Uyar, Haci Halil Uslucan, Stefan Becker, Bernhard Ferdinand Henning

**Affiliations:** 1 Central Patient Admission Unit/Emergency Department, Marienhospital Herne, Ruhr-University Bochum Medical Centre, Hölkeskampring 40, Herne 44625, Germany; 2 Stiftung Zentrum für Türkeistudien und Integrationsforschung, Altendorfer Straße 3, Essen 45127, Germany; 3 Department of Internal Medicine I, Marienhospital Herne, Ruhr-University Bochum Medical Centre, Hölkeskampring 40, Herne 44625, Germany

**Keywords:** Language barrier, Language skills, Turkish migration background, Turkish migrants, Germany, University hospital, Translation, Interpreter, Provision of information, Invasive procedures, Consent to treatment, School education, Hospitalised patients

## Abstract

**Background:**

Today more than two million people with Turkish migration background live in Germany making them the largest ethnic minority in the country. Data concerning language skills and the perception of medical information in hospitalised patients with Turkish migration background (T) are scarce. Our study is the first to gather quantitative information on this important subject.

**Methods:**

T and hospitalised German patients without migration background (G) of our university hospital were prospectively included into a cross-sectional study and completed a questionnaire - each group in the appropriate language (T: Turkish, G: German).

**Results:**

121 T and 121 G were included. Groups significantly differed in age (T: 44.9 ± 17.8, G: 56.9 ± 16.7y) and proportion of males (T: 37.2, G: 54.5%) but not regarding the proportion of college graduates (T: 19.3, G: 15.7%). The majority of T was born in Turkey (71%) and is of Turkish nationality (66%). 74% of T speak mainly Turkish at home; however, 73% speak German at work. 74.4% of T self-rated their German linguistic proficiency as “average” or better while 25.6% reported it as “very bad” or “bad”. 10.7% of T need translation in order to pursue everyday activities. T were significantly less satisfied with the physician’s information on disease and estimated to understand significantly less of what the physician told them: 46.3% of T estimated their reception of the physician’s information to be “average” or worse. 43.3% of T had the impression that it would have helped them “much” or “very much” to be aided by an interpreter at the hospital. The information transmitted while giving informed consent to invasive medical procedure was judged to be “mostly” or “completely” sufficient by the majority of T (76%) and G (89.8%). In this setting 37 of 96 T (38.5%) reported being helped by an interpreter – in most cases (64.9%) a family member.

**Conclusion:**

Although the majority of patients with Turkish migration background have spent most of their lives in Germany (28.94 ± 10.41y) a large part of this population has limited German language skills and difficulties obtaining medical information when hospitalised.

## Background

One of the principles of modern Western medicine is the physician acting to the patient’s benefit after obtaining informed consent. The physician thus has to make sure that the patient has the ability to understand the relevant information, to grasp the medical consequences of the situation, to weigh up different treatment options and to communicate a choice [1]. Ensuring an adequate exchange of information not only is a premise for diagnosis and therapy, but may also keep juridical problems at bay.

Language barriers between patients and their care providers inflict an array of problems. Insufficient language abilities may lead to discrimination by a healthcare system [2], to an increase in drug complications [3] or to a decreased use of preventive services [4].

To overcome language barriers physicians have to rely on the help of interpreters. Professional medical interpreters would be most suitable for this purpose [5]. However, in everyday practice healthcare providers may experience difficulties in obtaining an adequate interpreter [6] and often have to consider themselves lucky if they can find anybody able to help with translation. This might be a doctor, a nurse, somebody of the hospital staff, a patient’s relative or even another patient. The wrong choice of interpreter might not only lead to misinterpretation but also to social conflicts or an infringement of medical confidentiality.

Our hospital is located in the Ruhr-Area in the German state North Rhine – Westphalia (NRW). This region had been dominated by a thriving and labor-intensive coal mining and steel industry until the 1980s. Following a bilateral agreement between Turkey and Germany in 1961 many Turkish workers emigrated to this region [7]. In 1996 35% of the 2.014 million people of Turkish origin in Germany (hence 23.1% of all Turks living in the European Union) lived in NRW [8].

In our hospital patients with Turkish migration background (T) make up the largest ethnic minority. Currently a list of Turkish-speaking members of the hospital staff is available to find an interpreter more quickly. Courses in “medical Turkish” are offered for free to every employee. But further efforts to ameliorate the situation of Turkish speaking patients are to be taken since the above-mentioned measures have not lead to a perceptible improvement in everyday practice. However, it is difficult to overcome the language barrier with a tailored solution as long as the extent of the problem is not known. Our study is the first to evaluate and quantify language skills and perception of medical information in patients with a migration background in a German hospital.

## Methods

We conducted a prospective study of hospitalised patients at the University Hospital Marienhospital Herne, Germany. 121 T and 121 German patients without Turkish migration background (G) filled in a questionnaire during their hospital stay.

### Study population

The University Hospital Marienhospital Herne is part of the medical centre of the Ruhr – University Bochum, Germany. It is a 575 bed tertiary hospital with more than 20,000 admissions per year. The study was carried out at the larger of the two sites of the hospital with 362 beds. The following medical departments are located at this site: Internal Medicine, Nephrology, Pulmonology and Sleep Medicine, Gastroenterology, Cardiology and Angiology, Anaesthesiology, Oncology and Haematology, Geriatrics and Early Rehabilitation, General- and Abdominal Surgery, Vascular Surgery, Hand Surgery, Radiotherapy, Radiology, Gynaecology and Obstetrics including a unit specialised in diseases of the breast, the Newborn-Ward, the Interdisciplinary Intensive Care Unit, Central Patients Admission Unit and Palliative Care.

### Inclusion criteria

Adult patients (aged ≥18 years) were prospectively included into the study between the second and third inpatient day. Informed consent was a precondition for participation. Patients were defined as having a Turkish migration background if they had migrated from Turkey to the current territory of the Federal Republic of Germany after 1949 or were born in Germany as Turkish citizens or were born in Germany as German Citizens but had at least one parent who migrated from Turkey after 1949 or was born as a Turkish Citizen in Germany [9]. T were identified among hospital admissions by an onomastic method [10] according to Sauer et al. [11]. Turkish migration background was confirmed before administration of the questionnaire. G served as controls since difficulties of communication are also frequently reported by native hospital patients and might not only be due to migration background or lacking language skills. A formalised assessment for dementia or cognitive deficits was not part of the study. However, patients under guardianship or subject to legal incapacitation as well as other patients lacking the competence to consent to study inclusion in a legally valid manner were not included into the study. For ethical reasons and patient comfort inclusion of patients was only done during weekdays at regular working hours (8 am to 6 pm) and did not interfere with medical diagnostics or treatment. To determine the sample size required for detecting a medium effect size with an Alpha error of 5% a prospective power calculation was performed [12] assuming a sigma of 1.2 in both groups. To obtain a power of 80% the inclusion of 92 patients in each group would be necessary.

### Development and administration of a questionnaire

A specific questionnaire was developed for a reliable quantification of the interaction between patients and physicians. If applicable the items of the questionnaire were shaded in 5 progressive grades to allow a more precise quantification and better comparability (e.g. 1 = very dissatisfied, 2 = dissatisfied, 3 = neither satisfied nor dissatisfied, 4 = satisfied, 5 = very satisfied). Two bilingual translators converted the questionnaire from the German into the Turkish language. After a third translator had consented to it the questionnaire was tested with regard to its comprehensibility in a pilot test with patients from each group (T, G) and optimized thereafter. The following features were included in both versions of the questionnaire: migration background, demographic features and school education. Other items vary according to the patient’s ethnicity: 3 (T) items assessed language preferences during different activities of daily life (see Table 1), 5 (T) or 1 (G) items assessed linguistic proficiency (see Table 2), 5 (T) or 4 (G) items assessed the interaction between patient and physician (see Table 3), 2 (T,G) items assessed the flow of information concerning diagnostic or therapeutic procedures (see Table 4) and 3 (T) items assessed translation aspects (see Table 5).

**Table 1:** Language most currently spoken by patients with Turkish migration background

	**All**	**Born G**	**Born T**
**At home**	**n = 121**	**n = 35**	**n = 86**
German: n (%)	10 (8.26)	6 (17.17)	4 (4.65)
Turkish: n (%)	90 (74.38)	19 (54.29)	71 (82.56)
German and Turkish: n (%)	18 (14.88)	10 (28.57)	8 (9.30)
Kurdish: n (%)	3 (2.48)	0 (0)	3 (3.49)
**During leisure activities**	**n =121**	**n = 35**	**n = 86**
German: n (%)	23 (19.01)	17 (48.57)	6 (6.98)
Turkish: n (%)	82 (67.04)	12 (34.29)	70 (81.40)
German and Turkish: n (%)	14 (11.57)	6 (17.14)	8 (9.3)
Kurdish: n (%)	2 (1.65)	0 (0)	2 (2.33)
At work*	**n =96**	**n = 35**	**n = 61**
German: n (%)	73 (76.04)	30 (85.71)	43 (70.49)
Turkish: n (%)	17 (17.71)	2 (5.71)	15 (24.59)
German and Turkish: n (%)	6 (6.25)	3 (8.57)	3 (4.92)
Kurdish: n (%)	0 (0)	0 (0)	0 (0)

Results depicted as n (%). born *T*, Patients born in Turkey; *born G*, Patients born in Germany, *n*, Number * = not all of the T interviewed are working.

**Table 2:** Self-rated German and Turkish linguistic proficiency and literacy

**German language** “How would you rate your German linguistic proficiency?”						
T n(%):	**1:** 9(7.44)	**2:** 22(18.18)	**3:** 36(29.75)	**4:** 28(23.14)	**5:** 26 (21.49)	mean ± SD: 3.33 ± 1.21*^a^
**Turkish language** “How would you rate your Turkish linguistic proficiency?”						
T n(%):	**1:** 1(0.83)	**2:** 3(2.48)	**3:** 22(18.18)	**4:** 66(54.55)	**5:** 29(23.97)	mean ± SD: 3.98 ± 0.77*^a^
**German language in daily activities** “Is your German sufficient for your everyday-life activities?”						
T n(%):	**1**^**§**^**:** 13(10.74)	**2:** 22(18.18)	**3:** 23(19.01)	**4:** 22(18.18)	**5:** 41(33.88)	mean ± SD: 3.46 ± 1.39
**German literacy** “How well can you read and write in German?”						
T n(%):	**1:** 27(22.31)	**2:** 13(10.74)	**3:** 24(19.84)	**4:** 24(19.84)	**5:** 33(27.27)	mean ± SD: 3.19 ± 1.50*^b,^ *^c^
G n(%):	**1:** 0(0)	**2:** 0(0)	**3:** 9(7.44)	**4:** 49(40.50)	**5:** 63(52.07)	mean ± SD: 4.45 ± 0.63*^b^
**Turkish literacy** “How well can you read and write in Turkish?”						
T n(%):	**1:** 16(13.22)	**2:** 6(4.96)	**3:** 18(14.88)	**4:** 49(40.45)	**5:** 32(26.45)	mean ± SD: 3.62 ± 1.29*^c^

*n*, Number; *SD*, Standard deviation; *T*, Patients with Turkish migration background (n = 121). *G*, German patients without migration background (n = 121). Significant differences: *^a^: p < 0.0001, *^b^: p < 0.0001, *^c^: p = 0.0176. Rating: 1 = very bad, 2 = bad, 3 = average, 4 = good, 5 = very good. 1^§^ = very bad/only with the help of a translator.

**Table 3:** Interaction between patient and physician in charge

**Information on disease** “How satisfied were you about the physician in charge informing you about your disease?”						
T (n = 121) n(%):	**1:** 3(2.48)	**2:** 8(6.61)	**3:** 29(23.97)	**4:** 61(50.41)	**5:** 20(16.53)	mean ± SD: 3.71 ± 0.90*^d^
G (n = 119) n(%):	**1:** 2(1.68)	**2:** 2(1.68)	**3:** 17(14.29)	**4:** 65(54.62)	**5:** 33(27.73)	mean ± SD: 4.05 ± 0.80*^d^
**Information on diagnostics and treatment** “How satisfied were you about the physician in charge informing you about diagnostic and therapeutic measures to be taken?”						
T (n = 121) n(%):	**1:** 2(1.65)	**2:** 4(3.31)	**3:** 23(19.01)	**4:** 68(56.20)	**5:** 24(19.84)	mean ± SD: 3.89 ± 0.81
G (n = 119) n(%):	**1:** 3(2.52)	**2:** 3(2.52)	**3:** 14(11.77)	**4:** 62(52.10)	**5:** 37 (31.09)	mean ± SD: 4.07 ± 0.87
**Reaction to questions** “How well did the physician answer your questions?						
T (n = 119) n(%):	**1:** 1(0.84)	**2:** 11(9.24)	**3:** 26(21.85)	**4:** 47(39.50)	**5:** 34(28.57)	mean ± SD: 3.86 ± 0.96
G (n = 119) n(%):	**1:** 1 (0.84)	**2:** 1 (0.84)	**3:** 32(26.89)	**4:** 59(49.58)	**5:** 26(21.85)	mean ± SD: 3.91 ± 0.77
**Reception of information** “How well did you understand what the physician told you?”						
T (n = 121) n(%):	**1:** 11(9.09)	**2:** 13(10.74)	**3:** 32(26.45)	**4:** 30(24.80)	**5:** 35(28.93)	mean ± SD: 3.54 ± 1.26*^e^
G (n = 119) n(%):	**1:** 1(0.84)	**2:** 0(0)	**3:** 15(12.61)	**4:** 52(43.80)	**5:** 51(42.86)	mean ± SD: 4.28 ± 0.74*^e^
**Need for translation** “How much would it have helped if somebody had translated the physicians words into your language?”						
T (n = 121) n(%):	**1:** 41(33.88)	**2:** 14(11.58)	**3:** 5(4.13)	**4:** 19(15.70)	**5:** 42(34.71)	mean ± SD: 3.06 ± 1.74

*n*, Number; *SD*, Standard deviation; *T*, Patients with Turkish migration background. *G*, German patients without migration background. Significant differences: *^d^: p = 0.0029, *^e^: p < 0.0001 - for the remaining questions in Table 3 no significant differences between T and G could be detected (p > 0.05). Rating (information on disease and diagnostics and treatment): 1 = very unsatisfied, 2 = unsatisfied, 3 = neither satisfied nor unsatisfied, 4 = satisfied, 5 = very satisfied. (reaction to questions and reception of information): 1 = not at all, 2 = a little, 3 = mediocre, 4 = well, 5 = very well. (need for translation): 1 = not at all, 2 = a little, 3 = mediocre, 4 = much, 5 = very much.

**Table 4:** Obtaining informed consent for invasive diagnostic or therapeutic procedures

**Adequacy of information** “Did you feel sufficiently informed of the procedure?”						
T (n = 96) n(%):	**1**: 0(0)	**2:** 4(4.21)	**3:** 19(19.79)	**4:** 37(38.54)	**5:** 36(37.5)	mean ± SD: 4.09 ± 0.85*
G (n = 108) n(%):	**1:** 1(0.93)	**2:** 1(0.93)	**3:** 9(8.33)	**4:** 37(34.26)	**5:** 60(55.57)	mean ± SD: 4.43 ± 0.76*
**Possibility to ask questions** “Did you have enough opportunity to get answers to your questions about the procedure?”						
T (n = 95) n(%):	**1:** 0(0)	**2:** 4(4.21)	**3:** 22(23.16)	**4:** 30(31.58)	**5:** 39(41.05)	mean ± SD: 4.09 ± 0.90
G (n = 108) n(%):	**1:** 1(0.93)	**2:** 2(1.85)	**3:** 10(9.26)	**4:** 47(43.52)	**5:** 48(44.44)	mean ± SD: 4.29 ± 0.78

*n*, Number; *SD*, Standard deviation; *T*, Patients with Turkish migration background. *G*, German patients without migration background. * = significant difference (p < 0.05). Rating (information on disease and diagnostics and treatment): 1 = not at all, 2 = rather not, 3 = a bit, 4 = mostly, 5 = completely.

**Table 5:** Translation of information concerning invasive diagnostic or therapeutic procedures

**Interpreter** “Who did the translation of the physician’s information?” (responding patients n = 37)						
A family member n(%)^a^:	24 (64.87)					
A friend: n(%)^a^:	1 (2.70)					
Another patient n(%)^a^:	1 (2.70)					
A Turkish-speaking nurse n(%)^a^:	14 (37.84)					
A Turkish-speaking physician n(%)^a^:	6 (16.22)					
**Correctness and comprehensibility of translation** “Did you feel that the words of the physician were translated correctly and comprehensibly?”						
T (n = 37) n(%):	**1**: 0(0)	**2:** 0(0)	**3:** 4(10.81)	**4:** 18(48.65)	**5:** 15(40.54)	mean ± SD: 4.30 ± 0.65
**Additional benefit of a professional Interpreter** “How much better would a professional interpreter have helped you understand the Information given by the physician?”						
T (n = 59) n(%):	**1**: 34(57.63)	**2:** 10(16.95)	**3:** 4(6.78)	**4:** 5(8.48)	**5:** 6(10.17)	mean ± SD: 1.97 ± 1.38

*n*, Number; *SD*, Standard deviation. ^a^: It was possible to name more than one interpreter. The percentage therefore refers to all responding patients (n = 37). *T*, patients with Turkish migration background. Rating: 1 = not at all, 2 = a little, 3 = mediocre, 4 = much, 5 = very much.

After obtaining informed consent for study participation the questionnaire was completed by the study participants in the German (G) or Turkish (T) language depending on which group they belonged to. A bilingual person was available to help physically disabled or illiterate persons fill in the test.

### Analysis

Analyses were conducted using SPSS (version 20.0.0; SPSS Inc, Chicago, USA). To compare numerical data the Student T test was chosen. Categorical data were compared using the Fisher's exact test. A binary logistic regression analysis was performed to assess the effect of different explanatory variables on the quality of the reception of medical information by T (Question: “How well did you understand what the physician told you?”). For the purpose of the regression analysis we divided each of the relevant explanatory variables into two categories (patient’s nationality: German and Turkish-German vs. Turkish; patient’s country of birth: Turkey vs. Germany; language spoken at home or during leisure activities: German or Turkish and German vs. Turkish or Kurdish; German language and literacy, German language in daily activities as well as Turkish language and literacy: “very bad”, “bad” or “average” vs. “good” or “very good”. The response variable (quality of the reception of information) itself was categorised into “not at all”, “a little” and “mediocre” vs. “well” and “very well”. The study was approved by the ethics committee of the medical faculty of the Ruhr-University Bochum, Germany.

## Results

121 T and 121 G were included in the study during 7 months between July 2011 and March 2012. During this period 13,034 patients were admitted to the hospital, 877 (6.7%) of which were T according to an onomastic analysis. The demographic features and school education of both groups are shown in Table 6. The groups were significantly different in terms of age and sex. Age significantly differed in T depending on their place of birth (Turkey, n = 86, age: 51.98 ± 16.04, Germany, n = 35, age: 27.66 ± 5.55y, p < 0.0001) as well as the proportion of males/females (Turkey: 34/52, Germany: 11/24, p = 0.004). On average T have lived in Germany for 28.94 ± 10.41 years. For the patients born in Turkey the mean age at immigration to Germany was 21.79 ± 11.41 years. We have summarised different kinds of German school education if appropriate and made the following assignments: primary school = basic primary and secondary school (German: Volksschule), junior high school = secondary modern school (German: Hauptschule) and secondary high school for ages 10 to 16 (German: Realschule) and polytechnic upper school (German: Polytechnische Oberschule), high school = advanced technical college entrance qualification (German: Fachhochschulreife), college = A level (German: Abitur). The proportion of patients with a college degree did not significantly differ between the two groups (T: 19.3%, G: 15.7%).

**Table 6:** Demographic features and school education

	**T**	**G**	**Significance**
	**(n = 121)**	**(n = 121)**	
Age y ± SD	44.94 ± 17.70	56.93 ± 16.72	p <0.0001
Males n (%)	45 (37.2)	66 (54.5)	p =0.0097
**Patient’s nationality**			
German n (%)	37 (30.58)	121 (100)	
Binational^#^ n (%)	4 (3.30)	0 (0)	
Turkish n (%)	80 (66.12)	0 (0)	
**Patient’s birthplace**			
Germany n (%)	35 (28.93)	121 (100)	
Turkey n (%)	86 (71.07)	0 (0)	
**Highest school education**^**a**^	**(n = 109)**	**(n = 121)**	
Primary school	15 (13.76)	12 (9.92)	
Junior high school	64 (58.72)	81 (66.94)	
High school	9 (8.26)	9 (7.44)	
College	21 (19.27)	19 (15.70)	p =0.491

*T*, Patients with Turkish migration background. G: German patients without migration background. *y*, Year; *SD*, Standard deviation; *n*, Number; *Binational*, Patients with a double (Turkish/German) nationality. n(%) relates to the group with the same migration background. ^a^ = not all patients responded to this question (T: n =109, G: n = 121); results are depicted as n(%).

Table 1 shows which language is predominantly spoken by T (all T, T born in Germany and T born in Turkey) at home, during leisure activities and at work. While the majority of T speaks Turkish at home and during leisure activities (74.4 and 67.0% respectively) 76% speak German at work. The proportion of T speaking German in any context is much higher among T born in Germany than among T born in Turkey.

The self-rated ability to communicate in German or Turkish (spoken and written or read) is depicted in Table 2. Even though only 7.4% of T rated their German linguistic proficiency as “very bad”, 10.7% reported to need an interpreter in order to pursue every-day activities. T self-rated their Turkish spoken language and literacy significantly better than their German proficiency in the same fields (spoken language: Turkish: 3.98 ± 0.77, German: 3.33 ± 1.21, p < 0.0001; literacy: Turkish: 3.62 ± 1.29, German: 3.19 ± 1.50. p = 0.0176).

Interaction between hospitalised patients and their physicians essentially takes place during daily rounds. Some of the questions used in our study are aimed at evaluating the patients’ perception of important aspects of the interaction with the physician. Table 3 summarises the answers. T were significantly less satisfied with the information given on their disease than G. Furthermore T had the impression of understanding significantly less of what the physician told them than their German counterparts. More than half of the T felt that an interpreter would have helped them “much” (15.7%) or “very much” (34.7%) to understand what the physician told them. A binary logistic regression analysis was performed to assess the effect of different explanatory variables on the quality of the reception of medical information by T (Question: “How well did you understand what the physician told you?”). The following explanatory variables were used as described in the methods section: nationality, country of birth, language spoken at home or during leisure activities, self-rated Turkish and German linguistic proficiency and highest school education. 92 T could be included in the analysis. As the result of the regression analysis self-rated German language skills (Question: “How would you rate your German linguistic proficiency”) was the only variable significantly and independently associated with the quality of the reception of medical information by T (p = 0.035, adjusted odds ratio: 11.29, 95% CI: 1.18 – 107.83). Among all T an average or worse self-rating of German language skills predicted an average or worse quality of reception of the physician’s information with a good sensitivity and specificity (sensitivity: 91.0%, specificity: 75.4%, PPV: 76.1%, NPV: 90.7%). None of the other above mentioned variables reached significance.

Obtaining informed consent to invasive medical procedure (e.g. sedation, surgery, percutaneous angiography or endoscopy) is mandatory in many countries. In Germany the legislation obliges the physician to make sure that the patient has fully understood all relevant information before consenting to the procedure. In general, information on medical procedures is done verbally with the help of a standard brochure that will be individualised to the patient’s situation with schematic drawings and handwritten comments and signed by patient and physician. In our study 96 T and 108 G reported having been informed about a relevant diagnostic or therapeutic procedure. The responses to our questions concerning patient information are summarised in Table 4. Significantly more T did not feel to be sufficiently informed compared to their German counterparts. The proportion of patients feeling “not at all” or “rather not” sufficiently informed was below 5% in both groups (T: 4.21%, G: 1.85%).

Among the 96 T that had been informed about a relevant diagnostic or therapeutic procedure (giving informed consent) 37 T reported that this was done with the help of an interpreter. Table 5 shows who did the translation and how the patients judged the correctness of it. The additional benefit of a professional interpreter was only assessed among the 59 T who were not informed with the help of an interpreter. 18.6% of those patients felt that a professional interpreter would have helped them “much” or “very much”.

Among 121 T 76 (62.8%) were female and 45 (37.2%) were male. Between T female and T male there was no statistical difference in terms of age (42.7 ± 16.3 vs. 48.7 ± 19.6 y, p = 0.09), proportion of immigrants (68.4% vs. 75.6%, p = 0.534) and lifetime spent in Germany (27.9 ± 10.1 vs. 30.7 ± 10.9 y, p = 0.173). Furthermore no statistically significant difference could be found concerning language preferences (German only vs. Turkish or other), self-rated German and Turkish linguistic proficiency and literacy, interaction between patient and physician in charge and being able to give informed consent.

Of the 121 T 86 were born in Turkey (T born T), 35 were born in Germany (T born G). We did a subgroup analysis to differentiate between T depending on their place of birth. Language preferences of T depending on their birthplace are summarised in Table 1. Table 7 shows the distribution of age and gender as well as the proportion of patients with a high-school diploma or college degree. T born T were significantly older than T born G (born T: 51.98 ± 16.13y, born G: 27.66 ± 5.63y, p < 0.0001), but the proportion of males was similar and without statistically significant differences. The proportion of patients with a high-school diploma or college degree is significantly lower among T born T compared to T born G (born T: 17.57%, born G: 48.57%, p = 0.001). Table 8 shows the results of the self -evaluation of Turkish and German language skills as well as important aspects of the interaction with the physician. No significant difference could be detected concerning the self-rated ability to talk, read or write in the Turkish language between T born T and T born G. However, there was a substantial and statistically significant difference in all aspects involving the German language. In these fields T born G estimated to be more competent that T born T.

**Table 7:** Subgroup - analysis by country of birth of patients with Turkish migration background concerning demographics and school education

**Features**	**T born T**	**T born G**	**Significance**
	**(n = 86)**	**(n = 35)**	
Mean age y ± SD	51.98 ± 16.13	27.66 ± 5.63	p < 0.0001
Males n (%)	34 (39.53)	11 (31.43)	p =0.534
^**a**^**School education**	**(n = 74)**	**(n = 35)**	
High school or college n(%)	13 (17.57)	17 (48.57)	p = 0.001

T = patients with Turkish migration background. born T = born in Turkey. born G: born in Germany. y = year. SD = standard deviation. n = number. n(%) relates to the group with the same birthplace. ^a^ = not all patients responded to this question (T born T n = 74); results are depicted as n(%).

**Table 8:** Subgroup - analysis by country of birth of patients with Turkish migration background concerning linguistic proficiency, literacy, comprehension

**Self-rated German and Turkish linguistic proficiency and literacy**							
**German language** “How would you rate your German linguistic proficiency?”							
T born T n(%):	**1:** 9(10.47)	**2:** 22(25.58)	**3:** 34(39.53)	**4:** 15(17.44)	**5:** 6(6.98)	mean ± SD: 2.85 ± 1.06	
							p < 0.0001
T born G n(%):	**1:** 0(0)	**2:** 0(0)	**3:** 2(5.71)	**4:** 13(37.14)	**5:** 20(57.14)	mean ± SD: 4.51 ± 0.61	
**Turkish language** “How would you rate your Turkish linguistic proficiency?”							
T born T n(%):	**1:** 0(0)	**2:** 3(3.49)	**3:** 18(20.93)	**4:** 46(53.49)	**5:** 19(22.09)	mean ± SD: 3.94 ± 0.76	
							p = 0.374
T born G n(%):	**1:** 1(2.86)	**2:** 0(0)	**3:** 4(11.43)	**4:** 20(57.14)	**5:** 10(28.57)	mean ± SD: 4.09 ± 0.82	
**German language in daily activities** “Is your German sufficient for your everyday-life activities?”							
T born T n(%):	**1**^**§**^**:** 13(15.12)	**2:** 22(25.58)	**3:** 23(26.74)	**4:** 13(15.12)	**5:** 15(17.44)	mean ± SD: 2.94 ± 1.21	
							p < 0.0001
T born G n(%):	**1**^**§**^**:** 0(0)	**2:** 0(0)	**3:** 0(0)	**4:** 9(25.71)	**5:** 26(74.29)	mean ± SD: 4.74 ± 0.44	
**German literacy** “How well can you read and write in German?”							
T born T n(%):	**1:** 27(31.40)	**2:** 13(15.12)	**3:** 23(26.74)	**4:** 15(17.44)	**5:** 8(9.30)	mean ± SD: 2.58 ± 1.34	
							p < 0.0001
T born G n(%):	**1:** 0(0)	**2:** 0(0)	**3:** 1(2.86)	**4:** 9(25.71)	**5:** 25(71.43)	mean ± SD: 4.69 ± 0.53	
**Turkish literacy** “How good can you read and write in Turkish?”							
T born T n(%):	**1:** 15(17.44)	**2:** 3(3.49)	**3:** 15(17.44)	**4:** 30(34.88)	**5:** 23(26.74)	mean ± SD: 3.50 ± 1.39	
							p = 0.110
T born G n(%):	**1:** 1(2.86)	**2:** 3(8.57)	**3:** 3(8.57)	**4:** 19(54.29)	**5:** 9(25.71)	mean ± SD: 3.91 ± 0.98	
Interaction between patient and physician in charge							
**Reception of information** “How well did you understand what the physician told you?”							
T born T n(%):	1*: 11(12.79)	2*: 13(15.12)	3*: 29(33.72)	4*: 19(22.09)	5*: 14(16.28)	mean ± SD: 3.14 ± 1.24	
							p < 0.0001
T born G n(%):	1*: 0(0)	2*: 0(0)	3*: 3(8.57)	4*: 11(31.43)	5*: 21(60.00)	mean ± SD: 4.51 ± 0.66	

*n*, Number; *SD*, Standard deviation; *T*, Patients with Turkish migration background, *born T*, Born in Turkey, *born G*, Born in Germany, Rating: 1 = very bad, 2 = bad, 3 = average, 4 = good, 5 = very good. 1^§^ = very bad/only with the help of a translator, 1* = not at all, 2* = a little, 3* = mediocre, 4* = well, 5* = very well.

## Discussion

For the practice of medicine a sufficient exchange of information between physician and patient is crucial. Not only does the physician have to be able to comprehend and correctly interpret the patient but he is also obliged to sufficiently inform his patient in order to allow him making his decisions in self-determination. A language barrier is one of many obstacles in such a therapeutic relationship. Problems arising from language barriers have been described in many countries [13-16]. They mainly affect immigrants. The extend of the language barrier as well as the appropriate strategy to overcome it may vary greatly depending on the political, economic and social context of the population in focus. In the present study from Germany T have lower self-rated German language skills (see Table 2), a reduced comprehension of medical information (see Table 3) and show a greater uncertainty in the face of medical procedures that need to be consented to compared to G (see Table 4).

Since our study is the first of its kind among German hospitalised patients it remains uncertain whether the ethnic composition of our sample is representative for other hospitals and situations. However, the findings of our current study go well with two German representative surveys among people with Turkish migration background conducted nationwide [17] and in the state of NRW [18]. They found a mean age of 42 y and 41.1 y (current study: 44.9 y). 72% and 74.3% of the people interviewed were born in Turkey (current study: 71.0%), 23% and 40% were of German nationality (current study: 30.58%), 24% and 27.4% had a college degree (current study: 19.27%). The interviews of the nationwide survey [17] were conducted in the language of choice. 66% of the participants chose the Turkish language which correlates with the self-rating of our T patients estimating their German language skills to be “average” or worse in 74% of cases.

A previous study that did not assess language capacities has shown that Turkish migration background is an independent predictor for an unsuccessful rehabilitation treatment of patients with psychological or psychosomatic disorders in Germany [19]. The worrying finding of our study that 46.3% of T have the impression that their understanding of information given by the physician was “mediocre” or worse (G: 13.5%, see Table 3) could be an explanation to an unsuccessful treatment. Extensive and adequate information by the physician is virtually a *sine qua non* for a successful therapy that is irrefutable from a legal point of view. It seems evident that overcoming language problems would be a first and important step toward better migrant health in Germany. In the current study the majority of T (50.4%) responding to that question would appreciate the help of a professional medical interpreter. Even though translation by a professional medical interpreter might also have its limitations [15] it is the best solution in most cases [5]. Two aspects of our study also point out to a professional interpreter as the best solution. Firstly in our study 37.8% of T benefiting from a translation had a nurse as an interpreter (see Table 5). Interestingly translation by nurses has been reported to lead to serious miscommunication in as much as 50% of cases [14]. Secondly about a third of T (33.1%) estimate their Turkish literacy to be average or worse (see Table 2). This means that using signs or labels in Turkish language to guide patients in the hospital and using Turkish forms in order to inform patients about diagnostic or therapeutic measures does not eliminate the need for skilled intepreters. Unfortunately until now professional medical interpreters are rarely available in Germany – largely because of reimbursement issues [20]. A promising initiative is a German project aimed at training intercultural health mediators from immigrant communities [21]. The results of our study could motivate further research aimed at identifying appropriate and cost-effective solutions to the above-mentioned problems.

The subgroup analysis comparing T depending on their place of birth (Turkey or Germany) depicted in Table 1 (language preferences), Table 7 (demographics and school education) and Table 8 (linguistic proficiency, literacy, comprehension) shows significant differences in a number of aspects. T born T are older compared to T born G. This might be due to the fact that people migrating from Turkey to Germany during the peak times of labour emigration to Western Europe in the late 1960s and early 1970s [7,22] have aged meanwhile. Older Turkish family members joining their relatives in Germany also contribute to the higher age. T being born in G are mostly offsprings of the first generation migrants who have attended German schools and have generally experienced a different socialisation than their parents. These facts are very likely the explanation to the differences in language use (see Table 1), language skills and comprehension (see Table 8) and the lower age of T born G. A nationwide survey in Germany among people with Turkish migration background fits very well to our results: people aged 15 to 29 estimated their German language skills markedly better than people above the age of 30 [17]. Among the T born T 16 (18.60%) are of German nationality and 2 (2.33%) are Turkish-German binationals whereas among T born G 21 (60%) are of German nationality and 2 (5.71%) are Turkish-German binationals. German nationality itself might be a predictor for superior German language skills compared to Turkish nationality. This was at least true for participants of the above-mentioned survey [17]. Another reason for better German language skills among T born G might be the higher percentage of patients with a high-school diploma or college degree. Physicians dealing with patients of Turkish migration background might gain valuable insights simply by asking about their place of birth.

There is evidence that gender has an influence on the integration of T into the German society. A transnational marriage to a T residing in Germany has been the most common motive for immigration among female T. Female Turkish immigrants are reported to reach a lower level of school education and the self-rated knowledge of the German language was lower among female T responding to a representative German national survey [17]. In a representative survey among T living in the German state of NRW 63% of the men self-rated their ability to understand spoken German as “good” or better compared to 53% of the women [18]. We therefore assessed language preferences and self-rated language skills in T with regard to gender. No significant difference could be detected in any of the aspects studied. This might be due to the similar school education of women and men in our study. However, subgroups were too small to exclude gender differences with sufficient power.

Conducting a study among patients of different ethnic background and talking different languages brings along some challenges that we tried to overcome with our study design. We opted for a questionnaire in Turkish for T and one in German for G. As a bilingual person was always available and as practically all T spoke Turkish we never experienced any language difficulties concerning the administration of the questionnaire. Even though we put a lot of care and expertise in the generation and translation of the questions – especially the grading of the answers – we are conscious of the fact that answers to the same questions in different languages may not be suited for a direct comparison [23]. Repetitive administration of the questionnaire to patients was not part of the current study. The assessment of communication skills was done only as a self-assessment by a paper-based interview which might be prone to bias due to anticipation and fears [24]. Other instruments to measure language skills (e.g. formalised oral or written exam) were not employed since they would not have been feasible in the chosen setting. For the above described reasons reliability and validity of our questionnaire remains to be determined by future studies. The questionnaire is available as Additional file 1 in English, German and Turkish.

## Conclusion

55.4% of hospitalised patients with Turkish migration background (T) self-rated their German linguistic proficiency to be “average” or worse. Asked how well they understood what the physician told them, 46.3% of T respond “mediocre” or worse. The ability to comprehend medical information correlates well with the self-rated German language proficiency. The majority (50.4%) of T would appreciate the help of a professional interpreter “much” or “very much”. T born in Germany generally have less difficulties communicating in the German language and estimate to have a better capacity to cope with all other situations in the hospital they were asked about compared to T born in Turkey.

## Competing interests

The authors declare that they have no competing interests.

## Authors’ contributions

AG conceived the study and its design, coordinated the study, drafted and revised the manuscript, performed the statistical analysis and contributed substantially to the interpretation of data. MU helped designing the study, translated the interview into the Turkish language, carried out the interviews, and was responsible for the acquisition of data and critical reading of the manuscript. HU participated in the conception and design of study as well as in the interpretation of data and critical revision of the manuscript, and translated the questionnaire into the Turkish language. SB contributed to the interpretation of data and the critical revision of the manuscript. BH participated in the conception of the study as well as its design and coordination and helped to draft the manuscript. All authors read and approved the final manuscript.

## Pre-publication history

The pre-publication history for this paper can be accessed here:

http://www.biomedcentral.com/1472-6963/13/196/prepub

## Supplementary Material

Additional file 1 Questionnaire used in the evaluation of language competences in hospitalised patients with Turkish migration background in Germany and in native German controls. Click here for file

## References

[B1] Appelbaum PS Clinical practice. Assessment of patients’ competence to consent to treatment N Engl J Med 2007 357 18 1834 1840 10.1056/NEJMcp074045 17978292

[B2] Weinick RM Krauss NA Racial/ethnic differences in children’s access to care Am J Publ Health 2000 90 11 1771 1774 10.2105/ajph.90.11.1771 PMC1446405 11076248

[B3] Gandhi TK Burstin HR Cook EF Puopolo AL Haas JS Brennan TA Bates DW Drug complications in outpatients J Gen Intern Med 2000 15 3 149 154 10.1046/j.1525-1497.2000.04199.x 10718894 PMC1495358

[B4] Woloshin S Schwartz LM Katz SJ Welch HG Is language a barrier to the use of preventive services? J Gen Intern Med 1997 12 8 472 477 10.1046/j.1525-1497.1997.00085.x 9276652 PMC1497155

[B5] Karliner LS Jacobs EA Chen AH Mutha S Do professional interpreters improve clinical care for patients with limited English proficiency? A systematic review of the literature Health Serv Res 2007 42 2 727 754 10.1111/j.1475-6773.2006.00629.x 17362215 PMC1955368

[B6] Hadziabdic E Heikkila K Albin B Hjelm K Problems and consequences in the use of professional interpreters: qualitative analysis of incidents from primary healthcare Nurs Inq 2011 18 3 253 261 10.1111/j.1440-1800.2011.00542.x 21790876

[B7] Country Profile Turkey http://www.bpb.de/system/files/pdf/VQCFF5.pdf

[B8] Turks in Europe: From a Garbled Image to the Complexity of Migrant Social Realityhttp://www.cie.ugent.be/umanco/umanco5.htm

[B9] Duschek K Weinmann J Boehm K Laue E Brueckner G Bundesamt S Leben in Deutschland Haushalte, Familien und Gesundheit - Ergebnisse des Mikrozensus 2005 2006 Wiesbaden: Statistisches Bundesamt 1 95

[B10] Reiss K Makarova N Spallek J Zeeb H Razum O Identification and sampling of people with migration background for epidemiological studies in germany Gesundheitswesen 2012 Epub Date 2012/08/31 10.1055/s-0032-1321768 22932826

[B11] Sauer M Halm D Sauer M, Halm D Methodik und Durchführung der Befragung Erfolge und Defizite der Integration türkeistämmiger Einwanderer Entwicklung der Lebenssituation 1999 bis 2008 2009 Wiesbaden: VS Verlag für Sozialwissenschaften 132 133

[B12] Lenth R Some practical guidelines for effective sample size determination Am Stat 2001 55 3 187 193 10.1198/000313001317098149

[B13] Hadziabdic E Heikkila K Albin B Hjelm K Migrants’ perceptions of using interpreters in health care Int Nurs Rev 2009 56 4 461 469 10.1111/j.1466-7657.2009.00738.x 19930075

[B14] Elderkin-Thompson V Silver RC Waitzkin H When nurses double as interpreters: a study of Spanish-speaking patients in a US primary care setting Soc Sci Med 2001 52 9 1343 1358 10.1016/S0277-9536(00)00234-3 11286360

[B15] Sleptsova M If the provision of information runs into the language barrier--about the cooperation with interpreters Ther Umsch 2007 64 10 575 579 10.1024/0040-5930.64.10.575 18214211

[B16] Li S Pearson D Escott S Language barriers within primary care consultations: an increasing challenge needing new solutions Educ Prim Care 2010 21 6 385 391 21144177 10.1080/14739879.2010.11493944

[B17] Deutsch-Türkische Lebens- und Wertewelten 2012 http://www.infogmbh.de/images/downloads/TiD-Wertewelten%202012_Studie%20in%20Auszgen.pdf23742458

[B18] Sauer M Ergebnisse der zwölften Mehrthemenbefragung 2011 Integrationsprozesse türkeistämmiger Migrantinnen und Migranten in Nordrhein-Westfalen Essen 2012 Germany: Stiftung Zentrum für Türkeistudien und Integrationsforschung 1 228

[B19] Mosko M Schneider J Koch U Schulz H Does a Turkish migration background influence treatment outcome? Results of a prospective inpatient healthcare study Psychother Psychosom Med Psychol 2008 58 3–4 176 182 18421658 10.1055/s-2008-1067352

[B20] Neuber H Foreign patients: trying to avoid solitary solutions Dtsch Arztebl 2005 102 10 A652 A654

[B21] Salman R Weyers S Koller T MiMi Project - With Migrants for Migrants Poverty and social exclusion in the WHO European Region: health systems respond 2010 Copenhagen, Denmark: WHO Regional Office for Europe 52 63

[B22] Akgunduz A Labor migration from Turkey to Western Europe. An analytical review from its commencement (early 60s) to the recruitment halt (1973–74) Rev Eur Migr Int 1995 11 1 153 177 12319592

[B23] Hilton A Skrutkowski M Translating instruments into other languages: development and testing processes Canc Nurs 2002 25 1 1 7 10.1097/00002820-200202000-00001 11838715

[B24] MacIntyre P Noels K Clément R Biases in self-ratings of second language proficiency: the role of language anxiety Lang Learn 1997 47 2 265 287 10.1111/0023-8333.81997008

