# Supplementary material for: How do hospitalised patients with Turkish migration background estimate their language skills and their comprehension of medical information – a prospective cross-sectional study and comparison to native patients in Germany to assess the language barrier and the need for translation
Source: BMC Health Serv Res. 2013 May 28;13:196. doi: 10.1186/1472-6963-13-196 (PMC3701570; doi:10.1186/1472-6963-13-196)
Supplement: Additional file 1 — Questionnaire used in the evaluation of language competences in hospitalised patients with Turkish migration background in Germany and in native German controls. [file 1472-6963-13-196-S1.pdf]

Questionnaire used in the evaluation of language competences in hospitalised patients with Turkish migration background in Germany and in native German controls.

Additional material to the article “How do hospitalised patients with Turkish migration background estimate their language skills and comprehension of medical information – a prospective cross-sectional study and comparison to native patients in Germany to assess the importance of the language barrier and the need for translation.”

Authors: Arnd Giese, Müberra Uyar, Haci Halil Uslucan, Stefan Becker, Bernhard Ferdinand Henning

Corresponding author: Dr. Arnd Giese
Zentrale Patientenaufnahme/ Central Patient Admission
Marienhospital Herne
Universitätsklinikum der Ruhr-Universität Bochum
Hölkeskampring 40
44625 Herne
Germany
Tel.: +49 (0)2323 499-1551
Fax: +49 (0)2323 499-394
Email: arnd.giese@rub.de

English version

Please note that an English version of the questionnaire was generated solely for the purpose of making the questions more understandable for an English-speaking readership and to facilitate translation into other languages.

1 Language preferences

1.1 Which language do you usually speak at home?

☐ German ☐ Turkish ☐other language: ___________

1.2 Which language do you usually speak during leisure activities?

☐ German ☐ Turkish ☐other language: ___________

1.3 Which language do you usually speak at work?

☐ German ☐ Turkish ☐other language: ___________

2 Self-rated German and Turkish linguistic proficiency and literacy

2.1 How would you rate your German linguistic proficiency?

very bad	bad	average	good	very good
1	2	3	4	5

2.2 How would you rate your Turkish linguistic proficiency?

very bad	bad	average	good	very good
1	2	3	4	5

2.3 Is your German sufficient for your everyday-life activities (working, shopping in German stores, dealing with German authorities, communicating with a physician)?

very bad (only with the help of a translator)	bad	average	good	very good
1	2	3	4	5

2.4 How well can you read and write in German?

very bad	bad	average	good	very good
1	2	3	4	5

2.5 How well can you read and write in Turkish?

very bad	bad	average	good	very good
1	2	3	4	5

3 Interaction between patient and physician in charge during rounds

3.1 How satisfied were you about the physician in charge informing you about your disease?

very unsatisfied	unsatisfied	neither satisfied nor unsatisfied	satisfied	very satisfied
1	2	3	4	5

3.2 How satisfied were you about the physician in charge informing you about diagnostic and therapeutic measures to be taken?

very unsatisfied	unsatisfied	neither satisfied nor unsatisfied	satisfied	very satisfied
1	2	3	4	5

3.3 How well did the physician answer your questions?

not at all	a little	mediocre	well	very well
1	2	3	4	5

3.4 How well did you understand what the physician told you?

not at all	a little	mediocre	well	very well
1	2	3	4	5

3.5 How much would it have helped you if somebody would have served as a German / Turkish interpreter?

not at all	a little	mediocre	well	very well
1	2	3	4	5

4 Obtaining informed consent for invasive diagnostic or therapeutic procedures

4.1 Did you feel sufficiently informed of the procedure?

not at all	a little	mediocre	much	very much
1	2	3	4	5

4.2 Did you have enough opportunity to get answers to your questions about the procedure?

not at all	a little	mediocre	much	very much
1	2	3	4	5

4.3 If somebody acted as an interpreter - who did the translation of the physician's information?

☐a family member

☐a friend

☐another patient

☐a Turkish-speaking nurse

☐a Turkish-speaking physician

4.3.1 Did you feel that the words of the physician were translated correctly and comprehensibly?

not at all	a little	mediocre	much	very much
1	2	3	4	5

4.4 If nobody acted as an interpreter - how much better would a professional interpreter have helped you understand the Information given by the physician?

not at all	a little	mediocre	much	very much
1	2	3	4	5

German version

Please note that only the questions 2.4, 3.1, 3.2, 3.3, 3.4, 4.1 and 4.2 were part of the questionnaire for native German controls without migration background. Including the remaining questions into the questionnaire below was done solely for the purpose of making the questions more understandable and to facilitate translation.

1 Language preferences

1.1 Welche Sprache sprechen Sie zuhause am häufigsten?

☐deutsch ☐türkisch ☐andere Sprache: _______________

1.2 Welche Sprache sprechen Sie in Ihrer Freizeit am häufigsten:

☐deutsch ☐türkisch ☐andere Sprache: _______________

1.3 Welche Sprache sprechen oder sprachen Sie bei der Arbeit am häufigsten?

☐deutsch ☐türkisch ☐andere Sprache: _______________

2 Self-rated German and Turkish linguistic proficiency and literacy

2.1 Wie schätzen Sie Ihre deutschen Sprachkenntnisse ein?

sehr schlecht	schlecht	mittelmäßig	gut	sehr gut
1	2	3	4	5

2.2 Wie gut schätzen Sie Ihre türkischen Sprachkenntnisse ein?

sehr schlecht	schlecht	mittelmäßig	gut	sehr gut
1	2	3	4	5

2.3 Reichen Ihre Deutschkenntnisse für die Erledigungen des Alltages (Arbeit, Einkäufe in deutschen Geschäften, Behörden, Arzt)

sehr schlecht (nur mit Übersetzer)	schlecht	mittelmäßig	gut	sehr gut
1	2	3	4	5

2.4 Wie gut können Sie deutsch lesen und schreiben?

sehr schlecht	schlecht	mittelmäßig	gut	sehr gut
1	2	3	4	5

2.5 Wie gut können Sie türkisch lesen und schreiben?

sehr schlecht	schlecht	mittelmäßig	gut	sehr gut
1	2	3	4	5

3 Interaction between patient and physician in charge during rounds

3.1 Wie zufrieden sind Sie damit, wie man Sie im Rahmen der Visiten (Arztbesuch im Zimmer) über Ihre Erkrankung informiert hat?

sehr unzufrieden	unzufrieden	weder zufrieden noch unzufrieden	zufrieden	sehr zufrieden
1	2	3	4	5

3.2 Wie zufrieden sind Sie damit, wie man Sie im Rahmen der Visiten (Arztbesuch im Zimmer) über anstehende Untersuchungen und Behandlungen informiert hat?

sehr unzufrieden	unzufrieden	weder zufrieden noch unzufrieden	zufrieden	sehr zufrieden
1	2	3	4	5

3.3 Wie sehr ist der Arzt auf Ihre Fragen eingegangen?

überhaupt nicht	wenig	mäßig viel	ziemlich viel	äußerst viel
1	2	3	4	5

3.4 Wie gut haben Sie verstanden, was der Arzt bei der Visite gesagt hat?

überhaupt nicht	eher nicht	halbwegs	überwiegend	völlig
1	2	3	4	5

3.5 Wie sehr hätte es ihnen geholfen, wenn jemand bei der Visite deutsch / türkisch übersetzt hätte?

überhaupt nicht	wenig	mäßig viel	ziemlich viel	äußerst viel
1	2	3	4	5

4 Obtaining informed consent for invasive diagnostic or therapeutic procedures

4.1 Falls bei Ihnen eine Aufklärung durch einen Arzt über eine Operation oder eine Untersuchung stattfand (z.B. Herzkatheter, Magen- oder Darmspiegelung, Narkose, Operation, Chemotherapie, Blutübertragung): Haben Sie sich durch die Aufklärung ausreichend aufgeklärt gefühlt?

überhaupt nicht	eher nicht	halbwegs	überwiegend	völlig
1	2	3	4	5

4.2 Hatten Sie im Rahmen des Aufklärungsgesprächs ausreichend Möglichkeit, Fragen beantwortet zu bekommen?

überhaupt nicht	eher nicht	halbwegs	überwiegend	völlig
1	2	3	4	5

4.3 Falls bei dem Aufklärungsgespräch jemand die Worte des Arztes für Sie übersetzt hat: Wer hat die Worte des Arztes Übersetzt?

□jemand aus meiner Familie

□jemand aus meinem Freundes- oder Bekanntenkreis

□ein anderer Patient

□eine türkischsprachige Schwester / Krankenpfleger

□eine türkischsprachiger Ärztin / Arzt

4.3.1 Hatten Sie das Gefühl, dass der Übersetzer die Worte des Arztes korrekt und verständlich wiedergegeben hat?

überhaupt nicht	eher nicht	halbwegs	überwiegend	völlig
1	2	3	4	5

4.4 Falls niemand die Worte des Arztes übersetzte: Wie viel mehr hätte ein professioneller Dolmetscher Ihnen helfen können, den Inhalt des Aufklärungsgesprächs zu verstehen?

überhaupt nicht	wenig	mäßig viel	ziemlich viel	äußerst viel
1	2	3	4	5

Turkish version

1 Language preferences

1.1 Evde çoğunlukla konuştuğunuz dil?

☐Almanca ☐Türkçe ☐Başka bir dil: _______________

1.2 Boş zamanlarınızda çoğunlukla hangi dili konuşuyorsunuz?

☐Almanca ☐Türkçe ☐Başka bir dil: _______________

1.3 Iş yerinde çoğunlukla hangi dili konuşuyorsunuz veya konuştunuz?

☐Almanca ☐Türkçe ☐Başka bir dil: _______________

2 Self-rated German and Turkish linguistic proficiency and literacy

2.1 Almanca bilginizi nasıl buluyorsunuz?

Çok kötü	Biraz kötü	Ne iyi ne kötü	Oldukça iyi	Çok iyi
1	2	3	4	5

2.2 Türkce bilginizi nasıl buluyorsunuz?

Çok kötü	Biraz kötü	Ne iyi ne kötü	Oldukça iyi	Çok iyi
1	2	3	4	5

2.3 Almancanız günlük yaşamınızda yeterli mi? (İşyerinde, alışverişte, Alman resmi dairelerinde, doktorda)

Tamamen yetersiz (Tercüman yardımı gerekiyor)	Biraz yetersiz	Orta derecede yeterli	Oldukça yeterli	Tümüyle yeterli
1	2	3	4	5

2.4 Almanca okuma- ve yazmanız nasıl?

Çok kötü	Biraz kötü	Ne iyi ne kötü	Oldukça iyi	Çok iyi
1	2	3	4	5

2.5 Türkce okuma- ve yazmanız nasıl?

Çok kötü	Biraz kötü	Ne iyi ne kötü	Oldukça iyi	Çok iyi
1	2	3	4	5

3 Interaction between patient and physician in charge during rounds

3.1 Hastanedeki doktorun / doktorların hastalığınızla ilgili bilgilendirmesinden ne kadar memnunsunuz?

Hiç memnun değilim	Çok az memnunum	Orta düzeyde memnunum	Oldukça memnunum	Çok memnunum
1	2	3	4	5

3.2 Hastanedeki doktorun / doktorların size uygulanacak muayene ve tedavi ile ilgili bilgilendirmesinden ne kadar memnunsunuz?

Hiç memnun değilim	Çok az memnunum	Orta düzeyde memnunum	Oldukça memnunum	Çok memnunum
1	2	3	4	5

3.3 Doktor / doktorlar sorularınızla ne kadar iyi ilgilendi / cevapladı?

Hiç	Çok az	Orta derecede	Oldukça	Çok
1	2	3	4	5

3.4 Hastanedeki doktorun söylediklerini ne kadar iyi anladınız?

Hiç anlamadım	Çok az anladım	Orta derecede anladım	Çoğunu anladım	Tümünü anladım
1	2	3	4	5

3.5 Doktorun söylediklerini biri size tercüme etseydi ne kadar yardım etmiş olurdu?

Hiç	Çok az	Orta derecede	Oldukça	Çok
1	2	3	4	5

4 Obtaining informed consent for invasive diagnostic or therapeutic procedures

4.1 Bir doktor tarafından ameliyat veya muayene icin size detaylı bilgi verildi mi (Örneğin: Kalp anjiyosu, Mide- veya bağırsak- endoskopisi, narkoz, ameliyat, kemoterapi, kan nakli)? Cevabınız evet ise: Size verilen bilgiler sizi tatmin etti mi?

Hiç	Çok az	Orta derecede	Oldukça	Çok
1	2	3	4	5

4.2 Detaylı bilgilendirme görüşmesi çerçevesinde sorularınıza yeterli cevap alabilme imkanı buldunuz mu?

Hiç	Çok az	Orta derecede	Çokça	Tamamen
1	2	3	4	5

4.3 Detaylı bilgi görüşmesinde biri size doktorun söylediklerini tercüme etti mi? Cevabınız evet ise: Doktorun söylediklerini kim tercüme etti?

□Ailemden biri

□Arkadaş- / tanıdık çevremden biri

□Başka bir hasta

□Türkçe konuşabilen bir hemşire / hastabakıcı

□Türkçe konuşabilen bir doktor

4.3.1 Tercüme eden kişinin doktorun sözlerini doğru ve anlaşılır tercüme ettiği kanaati sizde oluştu mu?

Hiç	Çok az	Orta derecede	Oldukça	Çok
1	2	3	4	5

4.4 Detaylı bilgi görüşmesinde biri size doktorun söylediklerini tercüme etti mi? Cevabınız hayır ise: Profesyonel bir tercüman size detaylı bilgi görüşmesinin içeriğini anlamada daha ne kadar yardım edebilirdi?

Hiç	Çok az	Orta derecede	Oldukça	Çok
1	2	3	4	5
